# International Medical Congress—Dental Section

**Published:** 1890-03

**Authors:** W. D. Miller


### International Medical Congress—Dental Section.

The following, just received from Dr. Miller, gives interesting information to those concerned in the arrangements being made for the dental section at Berlin.—Ed.

In response to a call of the organizing committee (Professors Virchow, von Bergmann and Waldeyer) fifty delegates from the various universities and medical societies of Germany met at Heidelberg on the 17th of September, 1888, to take steps in the organization of the congress. At the meeting it was decided that the congress should be held in Berlin beginning August 4th, and closing August 9th, 1890.

An organizing committee consisting of Profs. Drs. Virchow, von Bergmann, Leyden and Waldeyer was elected and a general secretary, Dr. Lassar, appointed.

Eighteen sections, including dental surgery, were organized, each with a special committee of nine members.

An international medico-scientific exhibition is to be connected with the congress. Statutes and programme were adoped which will be given in as far as they particularly concern the dental section.

Art. II. " The Congress consists of physicians (approbirte Aerzte) who have registered their names and obtained their membership cards. Other savants who are interested in the work of the congress may be admitted as extraordinary members."

The delegates did not see fit to change this article so as to include dental surgeons, but decided that the article should be so interpreted as to admit dentists to membership. Since the meeting at Heidelberg the question has been raised whether dentists resident in Germany, but not possessing the German dental approbation (degree) could be admitted to membership. Regarding this point the chairman of the committee of organization decided that only those who possess the recognized degree of that country of which they were citizens, may be admitted to membership.

A German citizen holding only an American or Swiss degree is, therefore, not entitled to membership, no more is an American or English citizen not possessing the degree of his own country ; on the other hand, foreign citizens practicing in Germany are admitted without the German degree, provided they have the degree of their own country.

Members pay a fee of twenty marks and receive a copy of the transactions.

Art. III. The object of the Congress is exclusively scientific.

Art. X. All lectures and communications in the general sittings, or in those of the sections, must be handed, in writing, to the secretary before the close of the sitting. The editorial committee decides whether, or in what part, such commnications shall be included in the published transactions.

Art. XI. The official language of all sittings are German, English and French. Very short remarks may be made in other languages provided some member is prepared to translate them into one of the official languages.

Art. XII. Lectures are, as a rule, to be limited to twenty minutes, discussional remarks to ten minutes.

Art. XIV. Students of medicine and other persons, gentlemen and ladies, who are not physicians, but are interested in the proceedings of any particular session, may be invited by the president of that session, or on application, receive permission to attend as auditors.

There are to be no vice-presidents associated with the congress, but each section is empowered to elect a limited number of honorary presidents and a secretary for each of the official languages.

The committee of the dental section is composed as follows:

Busch, Berlin, Chairman; Cailas, Hamburg; Hesse, Leipzig; Fricke, Kiel; Hollander, Halle; Miller, Berlin; Partch, Breslav; Sauer, Berlin; Weil, Munich.

At a meeting of this committee, held on the 16th of October, 1889, it was decided that the hours from 9 to 12 A. M., should be devoted to practical demonstrations in the rooms of the dental institute, the demonstrations to consist of operations in filling, extraction and in mechanical dentistry, in short, operations in all branches of operative and mechanical dentistry.

Demonstrations in extraction and in artificial work are to be under the direction of Professor Busch; those in filling under that of Professor Miller. The theoretical exercises, etc., are to be held from 2 to 5. They will consist of the usual essays or lectures and the accompanying discussions; besides these, three subjects for general dsscussion are to be chosen, one to be introduced in the German language (on bromide of ethyl, by Professor Dr. Hollander), one in the English and one in the French languages.

Those desiring to deliver lectures or read essays on particular subjects are requested to send in, along with their announcement, a very short resume of the contents of the same.

Correspondence in German language to be directed to Professor Dr. Busch, Chairman, Dorstheen street, 40 Berlin; in French language, to Dr. Calais, Hohenleichen, 17 Hamburg; in English, to Professor Dr. Miller, Voss street 32 Berlin. In America, Drs. W. C. Barrett and J. Taft, H. J. McKollops; in Great Britain, Mr. J. H. Mummery, M. R. C. S., etc., and Mr. W. Bowman, Macleod, F. R. S., etc., on invitation by the committee, expressed their willingness to act in the capacity of honorary presidents. W. D. MILLER.

